# Risk Factors of HIV and Other Sexually Transmitted Infections in China: A Systematic Review of Reviews

**DOI:** 10.1371/journal.pone.0140426

**Published:** 2015-10-15

**Authors:** Yanping Zhao, Tongyong Luo, Joseph D. Tucker, William Chi Wai Wong

**Affiliations:** 1 Department of Family Medicine and Primary Care, Li Ka Shing Faculty of Medicine, The University of Hong Kong, Hong Kong S.A.R., China; 2 The Jockey Club School of Public Health and Primary Care, The Chinese University of Hong Kong, Hong Kong S.A.R., China; 3 University of North Carolina Project-China, Guangzhou, Guangdong Province, China; 4 London School of Hygiene and Tropical Medicine, London, United Kingdom; Centers for Disease Control and Prevention, UNITED STATES

## Abstract

**Background:**

Sexually Transmitted Infections (STIs) are a global challenge. China, once said to have eradicated STIs, is now facing a rapid rise in the prevalence of HIV/STIs. This review of reviews aims to map HIV/STI risk factors among the Chinese population, with the objective of identifying risk factors to inform the formulation of effective prevention strategies.

**Methods:**

A systematic search using key terms related to HIV/STIs, risk factors and the Chinese population in both English and Chinese databases (PubMed, PsycINFO, the Cochrane Library; Wanfang data, CNKI, VIP and SINOMED) was conducted, and peer-reviewed systematic reviews on the topic from 1991 to 2014 were selected. Identified risk factors were grouped into different level determinants based on the HIV Social Epidemiology Model, and then evaluated and reported based on the PRISMA checklist.

**Findings:**

Of the twenty-eight reviews included, the majority were focused on well-established, individual level risk factors within key populations, with some highlighting the complexity of interacting factors (e.g., alcohol use and higher income in male migrants). While twenty-two reviews covered individual factors, only ten mentioned social factors and five had contents on structural factors. There are gaps in the evidence on social and structural level impacts of HIV/STIs, such as on stigma, discrimination, health policy, access to care, and illicit drug control policies. Migration and social expectation appear to pose a significant threat in aggravating the HIV/STI situation in China; for example, incarceration patterns indicated a significant risk of HIV/STIs for female sex workers.

**Conclusions:**

Since international guidelines recommend an integrated and multi-level approach to HIV/STI prevention, a comprehensive approach targeting interventions at all levels along the continuum of care is needed to effectively curtail HIV/STI transmission in China. More research is needed to better understand the impact of socio-political interventions within a Chinese context.

## Introduction

Around the world, over one million people are infected with some kind of Sexually Transmitted Infection (STI) every day, making them a major global health problem [1]. The world’s most populous country, China, was said to have eradicated STIs 50 years ago but is now experiencing the most precipitous increase in the prevalence of STIs and facing a huge challenge of identifying how to control HIV and STIs [2, 3]. In 2012, 41,929 new cases of HIV/AIDS and about 780,000 People Living with HIV/AIDS (PLHIV) were reported in China [4]. Syphilis and gonorrhoea now represents the third and fifth most common category B infectious diseases in China. In 2012, there were about 1.6 million reported cases of syphilis, gonorrhoea and Hepatitis B in China [5, 6]. STIs are known to be associated with many serious health consequences including irreversible neurological problems, prematurity or stillbirth, cervicitis, pelvic inflammatory disease, chronic pelvic pain, infertility, and ectopic pregnancy in women [7–9].

A thorough understanding of the risk factors is a cornerstone for designing effective prevention and control interventions [10]. Thus far in China, many reviews have attempted to sum up the risks from various sources in different key populations such as men who have sex with men (MSM), or people who use drugs. Traditional disease prevention intervention often focuses on individual behaviours and overlooks social and contextual factors, some of which are believed to be the fundamental source of disease development [11]. Social epidemiology, which studies “the role of social factors in the aetiology of disease” has been used as a framework for the determinants of HIV, and is used as the underlying conceptual framework for this review [12]. The complexity of HIV/STIs requires a comprehensive socio-epidemiological approach to fully understand the interplay of different risk factors [13, 14]. Linking a social approach to epidemiology studies encourages a multidisciplinary approach which goes beyond the traditional individual behaviour method [15–18].

The aim of this study was to map out the risk factors for HIV/STIs in the Chinese population through a review of systematic reviews. It was anticipated that the overview of risk factors from pooled data could shed light on the priorities and inadequacy of the existing HIV/STIs studies in China and contribute to the fight against HIV/STIs globally.

## Methods

A comprehensive search protocol of peer-reviewed journals was developed. Three English databases (PubMed, PsycINFO, and the Cochrane Library) and four major Chinese databases (Wanfang Data, CNKI, VIP Chinese Journal Database, and SINOMED) were selected. HIV, syphilis and gonorrhoea only became reportable across China since 1991, and thus used as the start of the timeframe of this study (1991–2014) [19].

We included systematic reviews that studied the Chinese population, covering adult HIV/STI risk factors and including correlates of unsafe behaviour, prevalence and incidence. Exclusion criteria included: article published in languages other than English or Chinese; articles which were concerned with only congenital STIs; interventional studies; studies which examined non-Chinese populations. Search terms included three aspects: HIV/STIs (including Medical Subject Headings “sexually transmitted diseases”); risk factors relating to the framework (including prevalence, incidence as well as correlates of unsafe behaviours); and Chinese. Both qualitative and quantitative systematic reviews were included. The detailed search strategy can be found in S1 Appendix.

Two independent reviewers screened titles and abstracts of the search results, removing duplicates or ineligible reviews before proceeding to full text screening of eligible papers. Consensus was reached either through discussion or, if necessary, through a third researcher’s opinion. The raw data extraction sheet was compiled and modified independently to include basic information about the review based on the Social Epidemiology Framework, namely: the review period; database used; types of STIs; studied population; individual level risk factors (e.g. age), social level risk factors (e.g. networks) and structural factors (e.g. public policy); and limitations. The primary summary measures were the infection risk factors identified. The end point was HIV/STIs. Condom use was a proxy measure for avoiding HIV/STIs. Hepatitis C (HCV) was often included in the reviews as an STI—not only it was more prevalent in gay men with HIV but a lot of HCV patients were presented in the STI clinics. Additionally, HCV and HIV were often researched together. This study followed the Preferred Reporting Items for Systematic reviews and Meta-Analyses (PRISMA) guidelines (the checklist can be found in S2 Appendix). The quality of the studies was assessed according to the validated scale—Assessment of Multiple Systematic Reviews (AMSTAR) (Table 1) [20, 21].

**Table 1:** Quality assessment of included studies using the Assessment of Multiple Systematic Reviews (AMSTAR).

Study	SR/M	A1	A2	A3	A4	A5	A6	A7	A8	A9	A10	A11	AMSTAR
Chow et al. (2011)	M	0	1	1	0	0.5	1	1	0.5	1	1	0.5	**7.5**
Li et al. (2011)	M	0	1	1	0	0.5	1	1	0.5	1	1	0.5	**7.5**
He et al. (2011)	SR	0	1	1	1	0.5	1	0	0	1	1	0.5	**7**
Guo et al. (2011)	SR	0	0	1	0	0.5	1	0	0	1	0	0.5	**4**
Gao et al. (2009)	M	0	0	1	0	0.5	1	1	0	1	1	0	**5.5**
Yun et al. (2011)	M	0	1	1	0	0.5	1	1	1	1	1	0.5	**8**
Meng et al. (2013)	M	0	0.5	1	0	0.5	1	0	0	1	1	0.5	**5.5**
Chow et al. (2011)	M	0	0.5	1	0	0.5	1	1	0	1	1	0.5	**6.5**
Qiu et al. (2013)	M	0	0	1	1	0.5	0.5	0	0	1	1	0	**5**
Chow et al. (2012)	M	0	0.5	1	0	0.5	1	1	0	1	1	1	**7**
Zhuang et al. (2012)	M	0	1	1	0	0.5	1	1	1	1	1	1	**8.5**
Chow et al. (2011)	M	0	1	1	1	0.5	1	0	0	1	1	1	**7.5**
Zhuang et al. (2012)	M	0	1	0	0	0.5	1	1	1	1	1	1	**7.5**
Bao et al. (2009)	SR	0	0	0.5	0	0	0	0	0	1	0	1	**2.5**
Wang et al. (2010)	M	0	0	0.5	0	0.5	NA	0	0	1	0	0	**2**
Xing et al. (2013)	M	0	0	0.5	0	0.5	NA	1	1	1	1	0	**5**
Hong et al. (2008)	M	0	0	0.5	0	0.5	1	0	0	NA	0	1	**3**
Yang et al. (2013)	M	0	1	0.5	0	0.5	1	0.5	0	1	0	1	**5.5**
Poon et al. (2011)	SR	0	0	0.5	0	0.5	1	0	0	NA	0	1	**3**
Liu et al. (2012)	M	0	0	0.5	0	0.5	1	0	0	1	1	1	**5**
Zhang et al. (2013)	M	0	1	0.5	0	0.5	1	1	0	1	1	1	**7**
Zhang et al. (2013)	M	0	1	0.5	0	0.5	1	1	0	1	1	1	**7**
Zhang et al. (2013)	SR	0	1	1	1	0.5	1	0	0	1	0	1	**6.5**
Li et al. (2010)	SR	0	0	0.5	0	0.5	1	0	0	1	0	1	**4**
Lin et al. (2006)	SR	0	0	0.5	0	0.5	0	0	0	NA	0	1	**2**
Yang et al. (2005)	SR	0	0	1	1	0.5	1	0	0	1	0	0	**4.5**
Hong et al. (2012)	M	0	0	0.5	0	0	0	0	0	1	0	0	**1.5**
Zang et al. (2011)	M	0	1	1	1	0.5	1	0	0	1	0	1	**6.5**
Mean													**5.43**
Standard Deviation													**2.03**

(SR) Systematic review; (M) Meta-analysis; (A1…A11) AMSTAR Items (please find in the AMSTAR Checklist in the S3 Appendix), scored as 0 or 1; (AMSTAR) AMSTAR Score ranging from 0–11; (NA) Not Applicable

## Results

The initial selection process identified 47 out of 425 search results. The initial step was to include both Chinese populations from within and outside of mainland China but very few articles on the latter emerged. A handful of reviews consisting of a mixture of Chinese and other nationalities were excluded due to the lack of clear distinction between risk factors for the two groups. The full-text screening further excluded 19 duplicated reviews leaving a total of 25 English and 3 Chinese systematic reviews for final inclusion (S1 Fig). The reviews reported in the Chinese literature focuse more on HIV and the related risk factors in key populations only (e.g. drug users and MSM) while those in the English literature tended to be more diverse.

Of the final included papers, most reviews (N = 23) focused on key populations including: ten on MSM [22–31], one on Money Boys [32], five on drug users [33–37], three on female sex workers (FSW) [38–40], two on migrants [41, 42], one on long-distance truck drivers (LDTDs) [43], and one on combined high-risk groups [44]; three studies assessed key and general population [45–47], one on blood donors [48], and one in sero-discordant couples [49]. Different risk factors were extracted and put into a template. The majority of the risk factors identified were at the individual level rather than at the social or structural levels. A summary of the included studies can be found in S4 Appendix.

Based on the PRISMA checklist, the majority of the reviews identified themselves as a systematic review and/or meta-analysis in the title, with the exception of three [38, 40, 45]. Most studies presented structured abstracts, clear objectives and rationales but lacked detailed protocol or registration. Inclusion criteria were reported in all but one study [47]. Twenty-five reviews included details of the study selection; three did not [36, 38, 47]. All reviews mentioned the information sources and provided a full electronic search strategy, although few contacted the study authors to identify additional studies. The majority of the reviews reported the data collection process and items, though more than half failed to include the risk of bias in individual studies. Twenty-six studies stated limitations of the review, but two reviews did not [30, 36]. The median number of studies included in each review was 45 with an interquartile range of 40–92.

### Individual factors

Individual factors include individual characteristics, socioeconomic position and behaviours.

#### Individual characteristics

1. Younger age was a significant risk factor for STIs in four reviews [34, 40, 47, 48], but limited evidence was found for HIV. Among migrant returnees, STIs were higher among 18–30 years (8.7%) than among 31–45 years (2.7%) and 46–60 years (1.5%) [47, 50]. Being under 40 was associated with higher rates of HCV infection among entrants of methadone maintenance treatment (MMT) (<30 years OR = 1.88 (95% confidence interval (CI) 1.31–2.69); 30–40 years OR = 2.21 (95%CI 1.54–3.18) compared to >40 years) [34]. Sixty percent of HIV positive voluntary blood donors were below 30 years (compared to 40% 31–55 years) [48]. Chances of gonorrhoea/chlamydia co-infection among FSW under the age of 20 were 2.60 times (95% CI 1.53–4.44) of those above 20 [40, 51].
2. The prevalence of HIV/STIs differs by gender. Three reviews indicated a higher prevalence of STIs for women than men [42, 46, 47]. One review found that male drug users are one-and-a-half times more likely to be HIV affected in high prevalent areas (defined as >10,000 HIV infected drug users, i.e. Yunnan, Guizhou, Sichuan, Guangxi and Xinjiang) [34]. However, in low prevalence areas, female drug users were twice as likely to be HIV infected, with the odds of male-to-female infection being 0.46 (95%CI 0.27–0.79) [34].
  Migrants in cities had 6.70 times (95%CI 6.05–7.41) risk of contracting HIV compared to the general Chinese population, while female migrants were at even higher risk OR = 12.18 (95%CI 11.11–13.35) [42]. Compared to male migrants (4.2%, 95%CI 3.7–4.7%), females had higher STIs prevalence (14.1%, 95%CI 6.4–21.8%) [47, 50]. Female drug users had two-to-tenfold higher prevalence of syphilis than male drug users [46]. Differences in gender were not significant among drug users for viral STIs such as HIV/HBV/HCV [34, 36, 37].
3. Weak associations were found between marriage and HIV/STIs risks among different groups. One study found that unmarried migrants were more likely to engage in commercial sex (OR = 1.49; 95%CI 1.10–2.01) and become infected with STIs (OR = 1.56; 95%CI 1.26–1.93) compared to married migrants [41]. However, another review found that 80–85% of STIs patients were married [47].
4. Amongst drug users, ethnic minorities had higher rates of HIV infection (OR = 3.08; 95%CI 1.81–5.24) [37].
5. Three reviews highlighted importance of concurrent HIV/STIs as risk factors for acquiring other STIs [22, 23, 40]. For MSM, a positive correlation was observed between HIV and syphilis (2003–2008) [22]. Baseline syphilis infection was a risk factor for HIV infection, with Relative Risk (RR) of 3.33 (95%CI 1.97–5.62) [23]. HIV positive FSW were more likely to be infected with syphilis, with an Adjusted Odds Ratio (AOR) of 5.7 to 8.1 [40, 52, 53]. FSW with Herpes Simplex Virus type 2 (HSV-2) were more likely (OR = 2.2; 95%CI 1.05–4.70) to be infected with HIV and vice versa (AOR = 2.6; 95%CI 1.30–5.38) [40, 54, 55]. Surprisingly, FSW infected with trichomoniasis were much more likely (AOR = 11.2; 95%CI 2.9–42.7) to be infected with HIV and vice versa (AOR = 5.02; 95%CI 1.4–17.0) [40, 53]. Current HCV (OR = 5.9; 95%CI 2.1–15.9) or syphilis positive (AOR = 5.3; 95%CI 2.02–13.64) posed greater risks for acquiring HSV-2 among FSW [40, 54]. Though one review found that HIV was not associated with STIs history among drug users OR = 1.26 (95%CI 0.63–2.51) [37].

#### Socioeconomic positions

1. Low educational attainment increased HIV risks in certain populations. Having nine or less years of education was a risk factor for HIV among drug users (OR = 1.32; 95%CI 1.01–1.74) [37].
2. Some occupations increased risks for HIV/STIs [32, 38, 43, 44, 46]. Among LDTDs, the pooled prevalence estimates of HIV were 0.19% (95%CI 0.15–0.24%) with RR = 3.33 (95%CI 2.40–4.62) and syphilis at 0.86% (95%CI 0.70–1.06%) (1995–2010), with RR = 1.65 (95%CI 1.35–2.03) compared to the general population [43]. FSW had a higher HIV prevalence (0.36%; 95%CI 0.12–0.71%) than the general population [44], and Money Boys had a greater HIV risk (OR = 1.29; 95%CI 1.09–1.54) than general MSM [32]. Syphilis prevalence among “possible” FSW (employees at entertainment centres, etc. where commercial sex is not the primary service) was about 0.83% (95%CI 0.62–1.30%) compared to food and service employees (0.30%; 95%CI 0.20–0.50%) [46]. Longer duration of working in the sex work industry was associated with an increased risk of developing a syphilis infection in FSW (OR = 1.98; 95%CI 1.08–3.62) [38]. Unemployment and HIV infection were weakly associated among drug users (OR = 1.34; 95%CI 1.02–1.76) [37].
3. Higher income was associated with STIs among male migrants. Those with lower income were less likely to have multiple sexual partners (OR = 0.61; 95%CI 0.47–0.78) and be infected with STIs (OR = 0.56; 95%CI 0.44–0.70) compared with their higher income counterparts [41].

#### Behavioural factors

1. Having multiple sex partners was found to be a significant risk factor for HIV/STIs, with RR of 2.81 for HIV infection (95%CI 1.59–4.95) among MSM [23]. The number of male sex partners was also related to syphilis infection for MSM [24]. Among the general population, one review reported that the majority of STIs patients had multiple sexual partners within the previous year, with a mean of 6.2 and 7.2 for men and women respectively [47]. Engaging in sex with both genders was associated with 30% increase in HIV infection (OR = 1.30; 95%CI 1.04–1.62), but this correlation was not seen for syphilis [27].
2. Low rates of condom use were observed among general and key populations: only 10% of migrants reported consistent condom use, with nearly 40% having never used a condom [47]. Low rates of consistent condom use were observed among FSW with their stable partners (8–15%) and clients (13–54%) [38]. Similar findings were identified among drug users, with a reported rate of consistent condom use between 0–28%, and a range of 32–100% reported never having used a condom [47]. Low rates of consistent condom use were reported among MSM with regular, non-commercial/casual, and commercial partners with 23.3% (95%CI 11.25–42.1%), 39.0% (95%CI 28.8–50.3%) and 55.8% (95%CI 41.4–69.4%) in the last six months respectively [29].
3. Anal intercourse without the use of condoms (especially receptive anal sex (RR = 3.88; 95%CI 1.44–10.47)) was found to be a significant risk factor associated with HIV infection among MSM [23, 24]. HIV infection was associated with anal sex with a male partner in the past six months (OR = 3.18; 95%CI 1.59–6.37) [24, 56]. MSM populations also showed an increased prevalence of syphilis prevalence with a median of 14.56% (95%CI 10.61–18.7%) (2000–2005), which was significantly higher than other key populations, including FSW and FSW clients at 3.04% (95%CI 2.99–5.79%) [46].
4. Illicit drug use was associated with a higher prevalence of HIV/STIs [34–37, 40, 44, 46]. Median syphilis prevalence was higher in drug users at 6.81% (95%CI 5.01–11.17%) compared to 0.30% (95%CI 0.20–0.50%) among food and service employees [46]. Methamphetamine use was specifically associated with syphilis infections among FSW (AOR = 2.5; 95%CI 1.1–5.0) [40, 57]. Intravenous drug use was strongly associated with HIV infection among FSW (AOR = 8.0–9.1; 95%CI 2.1–4.67 to 17.55–30.3) [40, 53, 55] and posed a risk factor for HIV/HCV/HBV infection among all drug users [36]. HIV prevalence among People Who Inject Drugs (PWID) was 12.55% (95%CI 12.25–12.85%), with odds ranging from 3.73 to 4.29 fold higher than other drug users (prevalence = 1.05% (95%CI 0.95–1.16%)) [34, 37]. Similarly, HCV infection rates among PWID were ten times higher (OR = 10.82; 95%CI 7.60–15.40) than other drug users among MMT clinics entrants [34]. Long duration of drug use (>5 years) was positively correlated with increased HCV infections (OR = 2.69, 95%CI 1.07–6.78) [36].
5. Sharing of injecting equipment represented a great risk for HIV/HCV. HIV infection among needle sharers versus non-sharers ranged from OR = 2.47 (95%CI 1.44–4.23) to 4.46 (95%CI 2.71–7.34) whereas, for HCV, OR = 3.41 (95%CI 2.56–4.54) [34, 37].
6. Alcohol use was identified as a risk factor for STIs. A study of 16,797 female drinkers found that drinkers had an increased RR of 1.56 (95%CI 1.20–2.03) for trichomonas vaginalis compared with abstainers. Those who consumed 1–9 drinks per week or had an alcohol abusing partner displayed an increased risk of trichomonas infection (RR = 1.70, 95%CI 1.30–2.23 and OR = 2.53, p = 0.01) respectively [45, 58]. Frequent alcohol consumption in the past three months was associated with syphilis infection among MSM (OR = 1.9; 95%CI 1.1–3.2) [45, 59].

### Social factors

Social factors include social networks, neighbourhood effects, cultural context and social capital.

#### Social networks

1. Samples recruited from different networking sites showed different HIV infection rates among MSM [25, 26]. One review found higher HIV rates in saunas (3.6%-26.5%) than in gay bars (0.8%-10.3%) (OR = 3.1; 95%CI 2.0–5.0) [25]. In general, sampling by MSM networks found a higher HIV prevalence (2.8%, 95%CI 1.6 to 4.9%) when compared to snowballing and respondent-driven sampling (RDS) (2.1%, 95%CI 1.4 to 2.9%) [26].

#### Neighbourhood effects/geographic/physical environment

1. High heterogeneity of HIV prevalence was associated with the geographical locations of the studies. Overall, the highest HIV prevalence rates were found in southwest China within different key populations [31, 33, 44]. Provinces/regions with the highest HIV prevalence can be found in Table 2. A summary table of STIs could not be made based on geographical location as studies focused on different groups and STIs. Among MSM, the highest rates of syphilis were found in the northwest of China (14.2%, 95%CI 7.1–21.4%) [30]. Among drug users, the highest prevalence of HCV was found in South China (63.0%, 95%CI 49.7–76.2%), followed by Mid-China (58.9%, 95%CI 31.6–86.1%) (1994–2009) [36]. Among FSW, the highest prevalence of HSV-2 was in Yunnan (1996–2010) [40].
2. Working conditions are significantly associated with the prevalence of HIV/STIs among FSW [39, 40]. Those working in low-tier conditions (hair salons, massage parlours, small hotels or on the street) were more likely to be HIV-infected (OR = 2.0, 95%CI 1.12–3.47) [40, 55]. In medium and high-tier workplaces (star hotels, VIP clubs, big karaoke dancing bars, and saunas), HIV and syphilis prevalence were reported 0.32% (95%CI 0.16–0.48%) and 3.22% (95%CI 2.19–4.24%) respectively, while in low-tier workplaces were 0.39% (95%CI 0.18–0.61%) and 13.82% (95%CI 10.59–17.04%) respectively [39].

**Table 2:** Provinces/regions with the highest HIV prevalence amongst key populations in China.

Type of population Year	Provinces/regions with the highest prevalence						Pooled HIV prevalence
Blood donors 2010 [48]	Yunnan (125.97⁄100,000) Southwest	Guangxi (32.40⁄100,000) South	Guizhou (19.60⁄100,000) Southwest	Xinjiang (44.09⁄100,000) Northwest	Chongqing (18.22⁄100,000) Southwest	Tibet (24.02⁄100,000) Southwest	13.22⁄100,000 (12.10–4.40) (2000–2009)
FSW 2000–2011 [39]	Yunnan 4·79% (95%CI 3.35–6.24%) Southwest	Chongqing (0.98%; 95%CI 0.04–1.93%) Southwest	Guangxi (0.45%; 95%CI 0.31–0.59%) South	Sichuan (0.43%; 95%CI 0.20–0.66%) Southwest	Xinjiang (0·36%; 95%CI 0.18–0.54%) Northwest	Zhejiang (0.22%; 95%CI 0.10–0.34%) East Hainan (0.22%; 95%CI 0.06–0.37%) South	0.20% (95%CI 0.172–0.233%) (2000–2011)
MSM [28]	8.2% (95%CI 3.8–12.6%) in 2006 to 11.4% (95%CI 9.2–13.6%) in 2009 Southwest	3.9% (95%CI 0.2–7.5%) in 2006 to 8.6% (95%CI 7.1–10.1%) in 2009 Northeast	0.8% (95%CI 0.0–1.8%) in 2006 to 6.5% (95%CI 3.1–9.9%) in 2009 Northwest	1.2% (95%CI 0.2–2.3%) in 2004 to 5.4% (95%CI 3.3–7.5%) in 2009 South central	0.7% (95%CI 0.0–2.1%) in 2003 to 5.5% (95%CI 2.6–8.4%) in 2009 East	1.7% (95%CI 0.0–3.9%) in 2003 to 4.8% (95%CI 2.5–7.0%) in 2009 North	4.3% (95%CI 3.7–4.9%) (2003–2009) 0.6% (95%CI 0.0–2.1%) (2003) to 7.4% (95%CI 5.7–9.2%) (2009)
Drug users 1993–2009 [36]	12.9% (95%CI 7.7–18.2%) North	8.6% (95%CI 6.1–11.1%), Southwest	7.4% (95%CI 0.7–14.195%CI) Mid-China	3.0% (95%CI 2.3–3.6%) South	1.0% (95%CI 0.6–1.4%) East	0.9% (95%CI 0.2–1.7%) Northwest	3.3% (95%CI 2.9–3.7%)
PWID 2010 [44]	14·61% (95%CI 10.53–20.46%) Southwest	13.56% (95%CI 9.47–18.82%) Northwest	6·29% (95%CI 5.17–7.99%) South central				9.08% (95%CI 8.04–10.52%)

### Structural factors

Structural factors include: demographic changes; war and militarization; structural violence and discrimination; legal structures, and policy environment. No papers examining the risk of war and militarization, structural violence and discrimination were identified in the selected reviews.

#### Demographic change

1. Migration and mobility posed a significant risk factor for HIV infection. Compared to the general population, migrant workers recruited from urban areas had a 6.70 (95%CI 6.05–7.41) fold higher risk of HIV infection [42]. Furthermore, migrant MSM had relatively higher rates of HIV (4.3%, 95%CI 1.2–7.3%) than non-migrant MSM (1.1%, 95%CI 0–2.8%) [25].
2. Urbanization in China appears to contribute to the spread of HIV/STIs. Pooled estimates of HIV prevalence among migrants returning from urban areas was 0.18% (95%CI 0.12–0.29%), with the odds of HIV infection 3.16 (95%CI 2.06–4.84) times higher than the overall figure [42].

#### Legal structure

Prostitution is highly condemned in China, therefore sex workers are often detained for re-education through labour camps or analogous administrative detention [60]. A study reported higher HIV infection rates among FSW recruited from re-education centres (10.3%) compared to community-based FSW (0–1.4%) [38, 53]. A higher syphilis prevalence was found among imprisoned FSW ranging from 10.96% (95%CI 9.76–12.17%) to 12.49% (95%CI 4.95–17.8%) compared to possible FSW which ranged from 0.83% (95%CI 0.62–1.3%) to 3.34% (95%CI 3.10–3.59%) [39, 46].

#### Policy environment

1. For health policy and access to care, it is encouraging to see the positive impact of the National Free Antiviral Therapy (ART) project. Pooled HIV heterosexual infection rates among sero-discordant couples was 2.13 (95%CI 0.00–4.63) per 100 PY before the National Free ART project in 2003, dropping to 1.44 (95%CI 0.62–2.26) per 100 PY after implementation [49].

## Discussion

Based on the HIV social epidemiological framework, this systematic review of reviews provides a comprehensive picture on the risk factors of HIV/STIs among the Chinese population. On top of identifying and collating the important risk factors into different categories, this review highlighted the lack of consideration to important structural level determinants in the current literature, such as the impact of demographic changes and legal structure on the risk of HIV/STIs. Furthermore, research has mainly focused on key populations and individual level risk factors, neglecting social or structural level determinants. At the individual level, common risk factors emerged including individual characteristics (younger age, non-Han ethnic), behaviours (low condom use, sharing of intravenous equipment and anal sex) and socioeconomic position (lower education). Alcohol use and higher income in male migrants were identified as more complex interactive factors. Social networks and neighbourhood effects are significant risk factors presented at the social level.

The lack of structural level studies, such as those on stigma and discrimination, health policy and access to care, and illicit drug control policies, could be explained through multiple reasons. Presently, there is a lack of a clear definition of structural factors. There are no guidelines on how to conduct structural level interventions and limited information on the effectiveness of such interventions [12, 61]. However, even with limited evidence, it has been demonstrated that combining structural factors with an individual approach can significantly improve effectiveness of HIV prevention [62]. After the implementation of the National Free ART project in China, the pooled HIV heterosexual infection rate among sero-discordant couples dropped [49]. Internationally, the national 100% Condom Program, introduced in 1991, decreased STIs by 10 folds, and HIV incidence by 5 folds among the young Thai male conscripts from 1991–1993 to 1993–1995 [63].

Our review identified that among the structural risks, the incarceration pattern reveals a significant risk of HIV/STIs for FSW. Internationally, it is found that compared with the general public, the prevalence of HIV/STIs in the prison population is two-to-ten times higher, due to prevalence of unprotected sexual intercourse and intravenous drug use [64]. The objective of the re-education of sex workers, according to the Chinese policy, is to guide FSW away from “bad habits” [60]. However, evidence suggests the re-education policies are exposing FSW to increased risk of HIV/STIs, thus, there is need to re-consider the current policies [65]. Further, modelling suggests that decriminalization of sex workers could avert up to 33%-46% of HIV infections [66]. In China, more than 40 scholars and lawyers advocated for abolishment of detention education to the National People's Congress and the Chinese Political Consultative Conference in 2014 [67].

Most key populations (e.g. PWID, LDTD and FSW) were included in 1995 in the national surveillance system in China. Yet MSM was only added in 2002 [68], despite MSM studies accounting for more than one third of all reviews explored in this study. This is due to rapid increase of HIV infection in MSM in recent years in China from 1.77% (95%CI 1.26–2.57) in 2000 to 5.98% (95%CI 4.43–8.18) in 2010 [44]. Aligning with official reports, indicates that although HIV prevalence in key populations are stabilizing or decreasing, HIV infection among the estimated 3.1–6.3 millions of MSM have increased dramatically [4, 23–26, 28, 31, 69]. It has been identified that condom-less anal intercourse, multiple partners, migration and sex work were all found to pose significant risks for HIV infection among MSM. HIV prevention needs to take into consideration context-specific strategies. Engaging in sex with both male and female partners was associated with 30% increase in HIV infection, which presents a particular challenge in China given that at least one-third of Chinese MSM have wives or female partners to fulfil social expectations and pressures to marry and have children [27, 70]. In addition, a recent review found that although behavioural interventions alone can increase consistent condom use, it had little impact on HIV or syphilis infection [71], suggesting a need to incorporate behavioural, biomedical, social and structural dimensions [62].

This multi-level approach to HIV/STIs prevention and treatment is becoming increasingly important internationally [62]. The World Health Organization (WHO) recognizes the concurrent nature of vulnerabilities for key populations, thus recommends integrated service be provided [72]. For example, intravenous drug use is a significant individual risk factor for drug-using FSW to HIV/STIs infection [4, 34, 37, 40, 53, 55, 58]. A more holistic approach for an intervention would add opiate substitution treatment and needle and syringe programmes, to the traditional behavioural approaches [73]. WHO calls for screening, diagnosis and treatment of STIs to be offered routinely to key populations, “as part of comprehensive HIV prevention and care” [72]. Along with other international studies, the finding that co-infection is a risk factor for contracting HIV/STIs reinforces WHO guidelines suggests a need to scale up provider-initiated and voluntary testing services as well as counselling in STIs clinics in China [74, 75].

There are several limitations to this review. First, as most reviews contained data from cross-sectional studies, risk factors cannot infer causality. The pooled data nonetheless reinforces the associations of the risk factors and infections as the data is gathered systematically from all identified reviews. Secondly, as the review covers diverse sampling methods, population groups and STIs over a large time span, the overall picture might not reflect the real situation at a particular point in time. However, the review by Cai *et al*. concluded that no significant time trends were identified between 1980 and 2012, studying high-risk sexual behaviours through 174 observational studies [76]. Thirdly, most studies included were conducted in urban cities and might not represent all of China. Given the vast number of floating populations in China and the limited health facilities in rural areas, the rural situation of HIV/STIs may be equivalent if not worse than urban cities, thus demanding further investigation.

## Conclusion

It is a critical moment for China in terms of HIV/STIs control and prevention. A comprehensive picture of the risk factors, as presented in this review can help in effective planning of HIV/STIs prevention strategies. However, reviews on HIV/STIs risks among the Chinese populations are limited to individual factors. Though important, social and structural risk factors are desperately lacking, as highlighted by international guidelines and research for HIV/STIs prevention. A comprehensive approach targeting interventions at all levels, along the continuum of care is needed to effectively curtail HIV/STIs transmission in China. Our study recommends that more research is needed on the impact of socio-political interventions within a Chinese context.

## Supporting Information

S1 Appendix Search Strategy used for the different selected databases. (DOCX)

S2 Appendix The PRISMA checklist for identification of systematic reviews and meta-analysis. (DOCX)

S3 Appendix AMSTAR Checklist. (DOCX)

S4 Appendix A Table including a summary of all studies included in the review. (DOCX)

S1 Fig Flow chart of the study selection procedure of the review according to the PRISMA Flow Diagram 2009. (TIF)
