# Supplementary material for: Risk Factors of HIV and Other Sexually Transmitted Infections in China: A Systematic Review of Reviews
Source: PLoS One. 2015 Oct 15;10(10):e0140426. doi: 10.1371/journal.pone.0140426 (PMC4607362; doi:10.1371/journal.pone.0140426)
Supplement: S1 Appendix — (DOCX) [file pone.0140426.s001.docx]

**S1 Appendix 1 – Search Strategy used for the different selected databases**

**PsycINFO**

1. sexually transmitted disease or sexually transmitted infection or urethritis or non-gonorrhoeael or chlamydia infection or trachoma or gonorrhea or gonorrhea or gonorrhoeae or syphilis or treponema pallidum or HIV
2. risk factor or personal behavior or lifestyle or environmental exposure or epidemiological or predictor or impact or role or age or biological or demographic or geographic or behavioral or social proximate determinant
3. Chinese or China or Taiwan or Hong Kong or Macau or Macao or Singapore
4. 1+2+3 (895)
5. Systematic review (8)

**Cochrane**

1. sexually transmitted disease or sexually transmitted infection or urethritis or chlamydia infection or trachoma or gonorrhea or gonorrheas or gonorrhoeae or syphilis or treponema pallidum or HIV (TITLE, abstract, key word)
2. risk factor or personal behavior or lifestyle or environmental exposure or epidemiological or predictor or impact or role or age or biological or demographic or geographic or behavioral or social proximate determinant
3. Chinese or China or Taiwan or Hong Kong or Macau or Macao or Singapore (103)
4. Systematic review (99)

**Pubmed**

| Search **((sexually transmitted disease) AND (risk factor OR personal behavior OR lifestyle OR environmental exposure OR epidemiological OR predictor OR impact OR role OR age OR biological OR demographic OR geographic OR behavioral OR social proximate determinant)) AND (Chinese OR China OR Taiwan OR Hong Kong OR Macau OR Macao OR Singapore)** Filters: **Systematic Reviews** |
| --- |

Systematic review (103)

In parentheses are the numbers of studies found in each search.
