# Supplementary material for: Risk Factors of HIV and Other Sexually Transmitted Infections in China: A Systematic Review of Reviews
Source: PLoS One. 2015 Oct 15;10(10):e0140426. doi: 10.1371/journal.pone.0140426 (PMC4607362; doi:10.1371/journal.pone.0140426)
Supplement: S3 Appendix — (DOCX) [file pone.0140426.s003.docx]

**S3 Appendix 3 – AMSTAR Checklist**

AMSTAR Checklist*

A1. Was an 'a priori' design provided?

A2. Was there duplicate study selection and data extraction?

A3. Was a comprehensive literature search performed?

A4. Was the status of publication (i.e. grey literature) used as an inclusion criterion?

A5. Was a list of studies (included and excluded) provided?

A6. Were the characteristics of the included studies provided?

A7. Was the scientific quality of the included studies assessed and documented?

A8. Was the scientific quality of the included studies used appropriately in formulating conclusions?

A9.  Were the methods used to combine the findings of studies appropriate?

A10. Was the likelihood of publication bias assessed?

A11. Was the conflict of interest included?

Reference:

Shea BJ, Hamel C, Wells GA, Bouter LM, Kristjansson E, Grimshaw J, et al. AMSTAR is a reliable and valid measurement tool to assess the methodological quality of systematic reviews. Journal of clinical epidemiology. 2009;62(10):1013-20.

Shea BJ, Grimshaw JM, Wells GA, Boers M, Andersson N, Hamel C, et al. Development of AMSTAR: a measurement tool to assess the methodological quality of systematic reviews. BMC medical research methodology. 2007;7(1):10.
