# Supplementary material for: Risk Factors of HIV and Other Sexually Transmitted Infections in China: A Systematic Review of Reviews
Source: PLoS One. 2015 Oct 15;10(10):e0140426. doi: 10.1371/journal.pone.0140426 (PMC4607362; doi:10.1371/journal.pone.0140426)
Supplement: S4 Appendix — (DOCX) [file pone.0140426.s004.docx]

|  | | | **S4 Appendix 4 – A Table including a summary of all studies included in the review** | | | | | |
| --- | --- | --- | --- | --- | --- | --- | --- | --- |
| **1st author,**  **Years covered, reference No.** | **Journal name, published year**  **Language (only marked if not in English)** | **Studies covered**  **(how many pages)** | | **Population studied** | **Individual risk factors** | **Social risk factors** | **Structural risk factors** | **Funding** |
| Chow EP  2003–2010  [22] | PloS one.  2011 | 71  (11) | | MSM | Among MSM, a positive correlation (r=0.31, p=0.009) was observed between HIV and syphilis (2003–2008) |  |  | Australian Government Department of Health and Ageing; Australian Research Council (FT0991990); the University of New South Wales |
| Li HM  2005-2010 [23] | PloS one.  2011 | 12  (8) | | MSM | Syphilis infection was a risk factor for HIV infection with Relative Risk (RR) =3.33 (95%CI 1.97–5.62). Unprotected receptive anal intercourse in the past 6 months significant risk for HIV transmission in MSM (RR=3.88; 95%CI 1.44–10.47). Multiple sex partners among MSM a significant risk factor for HIV infection (RR=2.81; 95%CI 1.59–4.95) |  |  | Mega Project of China National Science Research for the 11th 5-Year Plan (2008ZX10001-005) WHO Project (UNICEF/UNDP/World Bank /WHO A70577), NIH Fogarty International Clinical Research Scholars Program (R24 TW007988) & the University of North Carolina (UNC) Center for AIDS Research |
| He Q  1999-2012  [24] | Southeast Asian journal of tropical medicine & public health  2013 | 45  (18) | | MSM | HIV infection associated with having anal sex with a male partner in the past 6 months (OR=3.18; 95%CI 1.59–6.37). The number of life-time male sex partners was correlated with syphilis infection among MSM |  |  | Partly supported by a Guangdong Provincial Medical Science Grant (A2004068, A2006068), CDC-GAP China Project and the Guangzhou Disease Control Project (2006-Zda-003) |
| Guo Y  2000-2009  [25] | AIDS and behavior  2011 | 33  (14) | | MSM |  | In Shenzhen, HIV infection rate was 3.6% in MSM recruited from saunas and 0.8% from gay bars. HIV infection in Chongqing was 26.5% among MSM from bathhouses and saunas vs. 10.3% among those from gay bars (OR=3.1; 95%CI 2.0–5·0). HIV among migrant MSM was 4·3% (95%CI 1·2–7·3) vs 1·1% (95%CI 0–2·8) among non-migrant MSM in 2008 | Migrant MSM had relatively higher rates of HIV (4.3%, 95%CI 1.2–7.3) than non-migrant MSM (1.1%, 95%CI 0–2.8) | NIH Research Grant R01NR10498 by the National Institute of Nursing Research and National Institute of Mental Health |
| Gao L  2001–2008  [26] | Sexually transmitted infections  2009 | 26  (5) | | MSM |  | Lower summarised prevalence (2.1%) for RDS & snowballing sampling method-based studies vs. prevalence of the studies sampling by VCT (3.8%) or MSM network 2.8% (95%CI 1.6 to 4.9) (p<0.05). |  | Not provided |
| Yun K  -2011  [27] | Sexually transmitted infections  2011 | 49  (8) | | MSM | Engaging in sex with both male and female partners was associated with 30% increase in HIV infection (OR=1.30; 95%CI 1.04–1.62), (this correlation not seen in syphilis) |  |  | Mega-projects of national science research for the 11th 5-Year Plan (2008ZX10001e001); National Nature Science Foundation of China (81001291); Shenyang Science and Technology Plan (F10-149-9-50) |
| Meng X  2003-2009  [28] | Sexual health  2013 | 83  (9) | | MSM | No difference in HIV prevalence between younger MSM < 25 and those older. |  |  | Not provided |
| Chow EP  2001-2010  [29] | BMC infectious diseases  2011 | 43  (17) | | MSM & their female partners | Consistent condom use with regular, non-commercial/casual, & commercial partners among MSM in the last 6 months were 23.3% (95%CI 11.25–42.1), 39.0% (95%CI28.8–50.3) 55.8% (95%CI41.4–69.4) respectively. |  |  | The Australian Government Department of Health and Ageing; the University of New South Wales; the World Bank Global HIV/AIDS Program; and grant no FT0991990 from the Australian Research Council |
| Qiu Y  2005-2012  [30] | Chinese Journal of AIDS & STD 2013  **In Chinese** | 82  (5) | | MSM |  | Among MSM, highest rates of syphilis in the northwest 14.2% (95%CI 7.1–21.4) compared to overall pooled prevalence 11.1% (95%CI 9.7–12.4). |  | Not provided |
| Chow EP  2001-2009  [31] | Sexually transmitted diseases  2011 | 94  (13) | | MSM |  | Southwest China had the  highest HIV prevalence (4.0%, 2.8%–6.5%) compared to other Chinese regions (2004-2006), which nearly tripled in 2007-2009 (still highest) |  | The Australian Government Department of Health & Ageing; the University of New South Wales; the World Bank Global HIV/AIDS Program; grant no 2008ZX10001-003 from the Ministry of Science and Technology in China; and grant no FT0991990 from the Australian Research Council |
| Chow EP  2000-2012  [32] | PloS one  2012 | 32  (16) | | Male sex workers (“Money boys”) | Male sex workers (“Money boys”) had a slightly higher risk (OR=1.29; 95%CI 1.09–1.54) of HIV infection than the broader MSM population. |  |  | The Australian Government Department of Health and Ageing; the University of New South Wales; the World Bank Global HIV/AIDS Program; and grant no. FT0991990 from the Australian Research Council |
| Zhuang X  2004-2010  [33] | BMC infectious diseases  2012 | 90  (15) | | People who use drugs (entrants to methadone maintenance treatment clinics) |  | Prevalence in HTAs were consistently higher than those in LTAs (HIV: 17.5% (95% CI: 14.0-21.6%) vs. 2.4% (95% CI: 1.6-3.5%) |  | Round 8 of the AusAID Australian Leadership Awards (ALA) Fellowships Program; Overseas Scholarship of Jiangsu Government, China; Nantong University, Jiangsu province, China; The University of New South Wales; Australian Research Council (FT0991990); Graduate student science and technology innovative project of Nantong University (YKC12035) |
| Zhuang X  2004-2011  [34] | Drug and alcohol dependence  2012 | 39  (10) | | People who use drugs (entrants to methadone maintenance treatment clinics) | Younger age (i.e. <40 years) was associated with higher rates of HCV infection among MMT entrants (<30 years OR=1.88 (95%CI 1.31–2.69); 30–40 years OR=2.21 (95%CI 1.54–3.18) compared to >40 years.  In HIV prevalent areas (>10,000 HIV+DU) such as Yunnan, Guizhou, Sichuan, Guangxi and Xinjiang, male drug users were 1.49 times (95%CI 1.11–1.99) more likely to be HIV infected. Differences in gender were not significant for HIV/HCV.  In low prevalence areas, the odds of male-to-female infection =0.46 (95%CI 0.27–0.79). Risk of HIV infection was higher among injectors> non-injectors (OR = 4.29, 2.70–6.79).  Similar patterns were also in HCV infection (injectors: OR = 10.82, 7.60–15.40).  HIV sharers > non-sharers (OR = 2.47, 1.44–4.23); HCV risk sharers: OR = 3.41, 2.56–4.54 than non-sharers |  |  | Round 8 of the Australian Leadership Awards (ALA) Fellowships Program; Overseas Scholarship of Jiangsu Government, China; New South Wales University, Australia; Nantong University, Jiangsu province, China |
| Bao YP  1997-2007  [35] | International journal of STD & AIDS  2009 | 40  (7) | | Drug users | HIV prevalence among IDU= 12.55% (12.25–12.85) non-IDU= 1.05% (0.95–1.16) |  |  | National Basic Research Program of China (No. 2009CB522007) & the 11th 5-Year Programme of the Chinese Ministry of Science and Technology (2007BAI07B04 & 2008ZX10001-003) |
| Wang H  1994-2009  [36] | Chinese Journal of Disease Control & Prevention 2010  **In Chinese** | 79  (5) | | Drug users | Gender differences not significant for HIV/HBV/HCV. Intravenous drug use was a risk factor for HIV/HCV/HBV infection. Long duration of drug use (>5 years) positively correlated with HCV infections OR=2.69 (95%CI 1.07–6.78). | Among DU, south China highest HCV prevalence at 63.0% (95%CI 49.7–76.2), mid-China 58.9% (95%CI 31.6–86.1), southwest China 52.5% (95%CI 33.7–71.4), north China 49.2% (95%CI 19.1–79.3) (1994–2009). |  | Not provided |
| Xing J  2002-2011  [37] | Disease Surveillance  2013  **In Chinese** | 14  (6) | | Drug users | Differences in gender were not significant for HIV. HIV infection not associated with marital status among DU. Among DU, ethnic minorities had higher rates of HIV infection (OR=3.08; 95%CI 1.81–5.24). HIV not associated with STI history among DU OR=1.26 (95%CI 0·63–2·51). <= 9 years of education was a risk factor for HIV infection among DU (OR=1.32; 95%CI 1.01–1.74).  Weak association between unemployment & HIV infection among DU (OR=1.34; 95%CI 1.02–1.76). HIV infection among PWID OR=3·73 (95%CI 2·95–4·70) than other drug users.  HIV infection among sharers than non-sharers OR= 4.46 (95%CI 2.71–7.34) |  |  | Not provided |
| Hong Y  1990-2006  [38] | AIDS behavior  2008 | 26  (14) | | FSW | Longer duration of sex work associated with syphilis infection in FSW (OR=1.98; 95%CI 1.08–3.62). FSW condom use with stable partners (8–15%) and clients (13–54%). |  | FSW in prison -a significant risk of HIV/STIs. A study reported higher HIV infection rates (10·3%) among women recruited from re-education centers vs. infection rates from the community FSW samples which 0 to 1·4% (mean 0·5%). | NIH Office of AIDS Research (R01MH064878-3S1) |
| Yang Z 2000-2011  [39] | PloS one  2013 | 190  (9) | | FSW |  | HIV and syphilis prevalence among FSW were 0.32% (95%CI 0.16–0.48) and 3.22% (95%CI 2.19–4.24) in medium and high-tier workplaces respectively and 0.39% (95%CI 0.18–0.61) and 13.82% (95%CI 10.59–17.04) in low-tier workplaces. | Higher syphilis prevalence among imprisoned FSW of 10.96% (95%CI 9.76–12.17) to 3.34% (95%CI 3.10–3.59). | National Major Scientific and Technological Special Project by China Ministry of Science and Technology  (2013ZX10004904-001 to NPW) |
| Poon AN  1996-2010  [40] | AIDS care.  2011 | 45  (21) | | FSW | Co-infection with gonorrhea & chlamydia among FSW < 20 were 2.6 times (95%CI 1.53–4.44) > those older than 20. HIV+ more likely to be infected with syphilis with AOR=5.7 (95%CI 1.6–20.7) to 8.1 (95%CI 1.1–68.5) & infected by any of the 3 STIs (gonorrhea, chlamydia, or trichomoniasis) were more likely to be infected with the other 2. Gonorrhea co-infection with chlamydia (64.9% 95%CI 57.9–71.3) more likely than chlamydia co-infected with gonorrhea (41.9% 95%CI 36.4–47.6). FSW with HSV-2 infection were twice (OR=2.2; 95%CI 1.05–4.70) as likely to be infected with HIV and vice versa (AOR=2.6; 95%CI 1.30–5.38). Infected with trichomoniasis more likely (AOR=11.2; 95%CI 2.9–42.7) to be infected with HIV & vice versa (AOR=5.02; 95%CI 1.4–17.0). Current infection with HCV (OR=5.9; 95%CI 2.1–15.9) or syphilis (AOR=5.3; 95%CI 2.02–13.64) posed greater risks for acquiring HSV-2 among FSW. Intravenous drug use strongly associated with HIV infection among FSW (AOR=8.0-9.1; 95%CI 2.1-4.67 to 17.55–30.3). Studies from Yunnan found high rates of HIV (28.2%) and syphilis (34.6%) among drug-using FSW. Methamphetamine use associated with syphilis infections among FSW (AOR=2.5; 95%CI 1.1–5.0). Drug-using FSW (60.8% intravenous drug using) with notably high prevalence of HCV (32.4%) and syphilis (18.6%) | Highest prevalence of HSV-2 infection among FSW was in Yunnan, -the data limited to 6 studies (1996–2010).  FSW working in low-tier conditions significantly more likely to be HIV-infected OR=2.0 (95%CI 1.12–3.47) |  | A cooperative agreement from the Centers for Disease Control and Prevention (CDC) through the Association of Schools of Public Health (ASPH) Grant Number CD300430 |
| Liu H  2000-2012  [41] | AIDS care.  2012 | 12  (9) | | Migrants | Unmarried migrants were more likely to engage in commercial sex (OR=1.49; 95%CI 1.10–2.01) and become infected with STIs (OR=1.56; 95%CI 1.26–1.93). For migrants, education not found to be related to sexual risks (commercial sex, multiple sexual partners, or infection with STIs). Male migrants with lower income less likely to have multiple sexual partners (OR=0.61; 95%CI 0.47–0.78) and be infected with STIs (OR=0.56; 95%CI 0.44–0.70) |  |  | National Social Science Foundation of China (08&ZD048, 09XSH005), Program for Changjiang Scholars and ]Innovative Research Team in Universities of the Ministry of Education of China (IRT0855). This study is also funded by the 985 Project of Xi’an Jiaotong University |
| Zhang L  2000-2011  [42] | Sexually transmitted diseases.  2013 | 54  (12) | | Migrants | Migrants in cities had 6·70 (95%CI 6·05–7·41) times higher risk of contracting HIV than the general Chinese population and female migrants were at even higher risk (OR=12·18; 95%CI 11·11–13·35). |  | Migrant workers recruited from urban areas had 6.70 (95%CI 6.05–7.41) times higher risk of HIV infection than that of the overall Chinese population. HIV prevalence among migrants returning from urban areas was 0.18% (95%CI 0.12–0.29), and the odds of HIV infection was 3.16 (95%CI 2.06–4.84) times higher than the rest of China | The universities of New South Wales (Sydney, Australia) and Bielefeld (Germany) and jointly by the Go8 Germany Joint Research Co-operation Scheme 2011 and the German Academic Exchange Service. The Kirby Institute is funded by the Australian Government, Department of Health and Ageing, and affiliated with the Faculty of Medicine, University of New South Wales |
| Zhang X. 1995-2011  [43] | International journal of infectious diseases  2013 | 46 HIV  23 Syphilis  (6) | | Long-distance truck drivers (LDTDs) | Pooled prevalence estimates of HIV and syphilis among LDTDs were 0.19% (95%CI 0.15–0.24) and 0.86% (95%CI 0.70–1.06) respectively (1995–2010), corresponding to RR=3.33 (95%CI 2.40–4.62) and RR=1.65 (95%CI 1.35–2.03) than general population. |  |  | The Australian Government Department of Health and Ageing, the University of New South Wales, the Endeavour Awards (2744_2012), the World Bank Global HIV/AIDS Program, grant number 2008ZX10001–003 from the Ministry of Science and Technology (China), FT0991990 from the Australian Research Council |
| Zhang L  1995-2010 [44] | The Lancet infectious diseases  2013 | 838  (9) | | IDU, FSW, MSM | FSW higher HIV prevalence 0·36% (95%CI 0·12–0·71) than that of the general Chinese population (0·05–0·06%). HIV more prevalent among People Who Inject Drugs (PWID) than all other drug users. | Southwest China had the highest HIV prevalence with different key populations. |  | The World Bank Group, the Australian Research Council, the University of New South Wales, and Chinese Center for Disease Control and Prevention |
| Li Q  1980-2008  [45] | AIDS behavior  2010 | 18  (10) | | MSM, FSW, & general population | A study of 16,797 female drinkers (aged 25–75) had an RR of 1.56 (95%CI 1.20–2.03) for trichomonas vaginalis vs. abstainers; those who had 1–9 drinks per week or with an alcohol abusing partner experience an increased risk of developing trichomonas infection with RR=1.70 (95%CI 1.30–2.23) & OR=2.53 (p=0.01) respectively. Quantitative hospital-based case-control studies reported positive associations between alcohol use and condyloma acuminata in STIs patients. Association between alcohol use and STIs in MSM inconsistent. Syphilis infection among MSM associated with frequent alcohol consumption in the past three months (OR=1.9; 95%CI 1.1–3.2). |  |  | Grant R01AA018090 from the National Institute on Alcohol Abuse and Alcoholism, National Institutes of Health |
| Lin C C  2000-2005  [46] | Sexually transmitted diseases  2006 | 174  (11) | | MSM, FSW & their clients, migrants, drug users, & general population. | Female drug users had 2-10 times higher prevalence of syphilis than male drug users. Syphilis prevalence among “possible” FSW was about 0.83% (95%CI 0.62–1.30) compared to food and service employees at 0.30% (95%CI 0.20–0.50) and commercial blood donors at 2.86% (95%CI 1.71–9.90).  MSM median syphilis prevalence was 14.56% (95%CI 10·61–18·7%) (2000-2005), highest median prevalence among all the groups studied, vs FSW and their clients at 3·04% (95%CI 2·99–5·79). Drug users 6·81% (95%CI 5·01–11·17) vs. 0·30% (95%CI 0·20–0·50) among food and service employees. |  | incarcerated FSW > possible FSWs 12.49% (95%CI 4.95–17.8) vs possible FSW at 0.83% (95%CI 0.62–1.3) | Fogarty-Ellison Fellowship for Global Health and the WHO Western Pacific Region |
| Yang H  1994-2004  [47] | Sexually transmitted diseases.  2005 | 29  (11) | | Drug users, former blood/ plasma donors, FSWs, attendees at STD clinics, rural-to-urban migrants, & general population. | STIs were higher among 18–30 years (8.7%) than 31–45 years (2.7%) and 46–60 years (1.5%). Female migrant workers had higher rates of STIs i.e. 14.1% (95%CI 6.4–21.8) vs male migrants 4.2% (95%CI 3.7–4.7). 80-85% of reported STIs cases were married. Condom use among DU (0%-28%), and reports of never having used a condom during 32% to 100%.  A hospital-based random sample from public STIs clinics in Shandong Province found most STIs patients had multiple sexual partners during the previous year, with a mean number of 6.2 partners for men and 7.2 for women. |  |  | Not provided |
| Hong Y  2000-2009  [48] | Tropical medicine &  international health  2012 | 87  (11) | | Voluntary blood donors | 60% of HIV positive voluntary blood donors were below 30 years of age (compared to 40% 31-55 years of age). | 23 of 29 provinces had prevalence< national average, and 6 provinces > national average: Yunnan (125.97 ⁄ 100 000), Guangxi (32.40 ⁄ 100 000), Guizhou (19.60 ⁄ 100 000), Xinjiang (44.09 ⁄ 100 000), Chongqing (18.22 ⁄ 100 000) and Tibet (24.02 ⁄ 100 000). |  | Not provided |
| Zang C  1992-2009 [49] | Chinese medical journal  2011 | 11  (7) | | General population (sero-discordant couples) |  |  | Pooled HIV heterosexual infection rate among sero-discordant couples before the National Free ART project in 2003 was 2.13 (95%CI 0.00–4.63) per 100 PY and after implementation dropped to 1.44 (95%CI 0.62–2.26) per 100 PY | The Fogarty International Center, NIH office of the Director, Office of AIDS Research, National Cancer Center, National Eye Institute, National Heart, Blood and Lung Institute, National Institute of Dental & Craniofacial Research, National Institute on Drug Abuse, National Institute of Mental Health, National Institute of Allergy and Infectious Diseases Health, through the International Clinical Research Fellows Program at Vanderblit (No.R24 TW007988) and the Mega-projects of Chinese National Science Research for the 11th Five-Year Plan (No 2008ZX10001-003) |

*Please note that empty cells mean that no such risk factors in that category were analysed in that study.
