# Supplementary figures and images for: Risk Factors of HIV and Other Sexually Transmitted Infections in China: A Systematic Review of Reviews
Source: PLoS One. 2015 Oct 15;10(10):e0140426. doi: 10.1371/journal.pone.0140426 (PMC4607362; doi:10.1371/journal.pone.0140426)

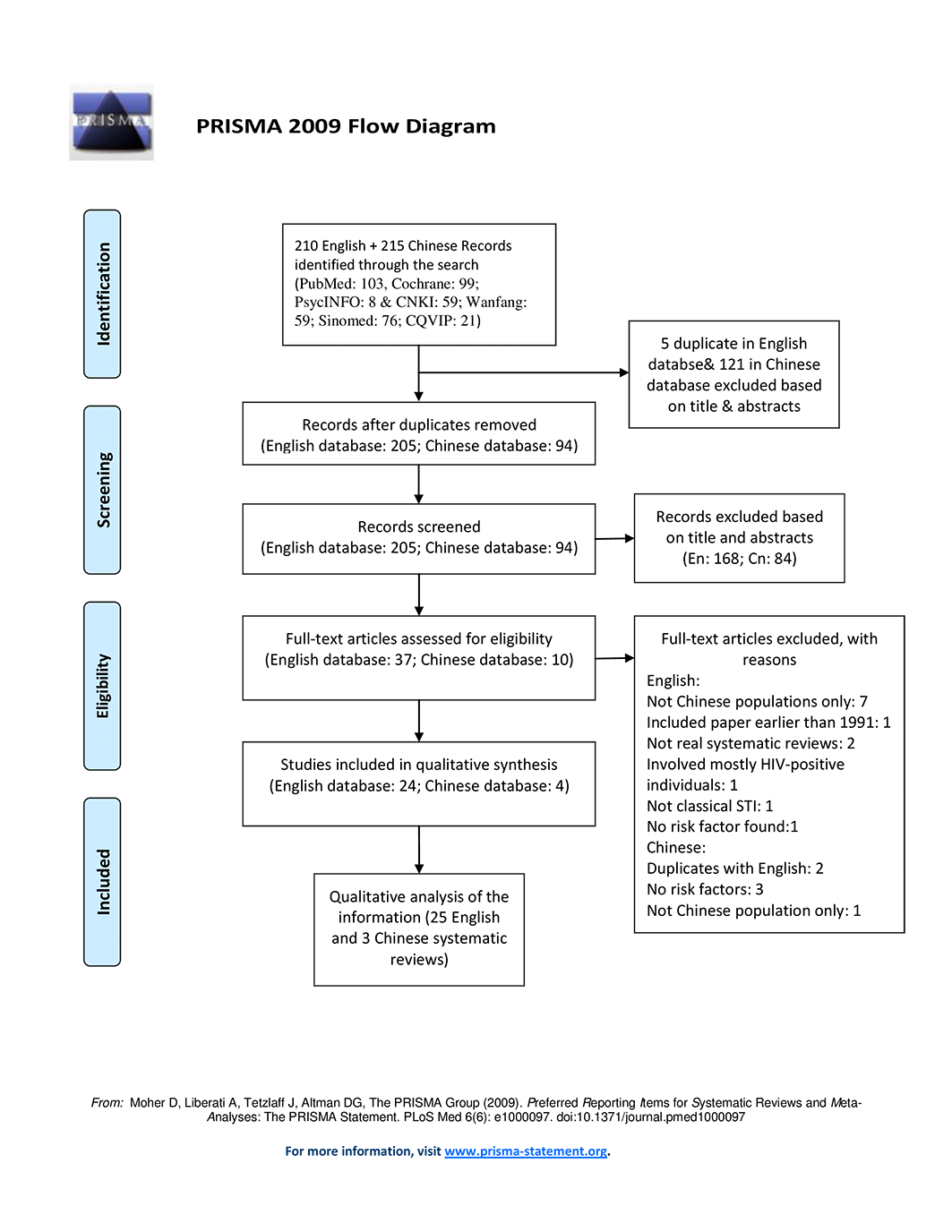

Supplement: S1 Fig — (TIF) [file pone.0140426.s005.tif]
